# Clinical Validity of a Prognostic Gene Expression Cluster-Based Model in Human Papillomavirus–Positive Oropharyngeal Carcinoma

**DOI:** 10.1200/PO.21.00094

**Published:** 2021-10-27

**Authors:** Stefano Cavalieri, Mara S. Serafini, Andrea Carenzo, Silvana Canevari, Ruud H. Brakenhoff, C. René Leemans, Irene H. Nauta, Frank Hoebers, Mari F. C. M. van den Hout, Kathrin Scheckenbach, Thomas K. Hoffmann, Laura Ardighieri, Tito Poli, Pasquale Quattrone, Laura D. Locati, Lisa Licitra, Loris De Cecco

**Affiliations:** ^1^ Head and Neck Medical Oncology Department, Fondazione IRCCS Istituto Nazionale dei Tumori, Milan, Italy; ^2^ Integrated Biology Platform, Department of Applied Research and Technology Development, Fondazione IRCCS Istituto Nazionale dei Tumori, Milan, Italy; ^3^ Fondazione IRCCS Istituto Nazionale dei Tumori, Milan, Italy; ^4^ Amsterdam UMC, Vrije Universiteit Amsterdam, Otolaryngology/Head and Neck Surgery, Cancer Center Amsterdam, the Netherlands; ^5^ Department of Radiation Oncology (MAASTRO), Research Institute GROW, Maastricht University, Maastricht, the Netherlands; ^6^ Department of Pathology, Research Institute GROW, Maastricht University Medical Center, Maastricht, the Netherlands; ^7^ Department of Otolaryngology, Medical Faculty, Heinrich Heine University Düsseldorf. Düsseldorf, Germany; ^8^ Department of Otorhinolaryngology Head and Neck Surgery, Ulm University Medical Center, Ulm, Germany; ^9^ Department of Pathology, ASST Spedali Civili of Brescia, Brescia, Italy; ^10^ Unit of Maxillofacial Surgery, Department of Medicine and Surgery, University of Parma—University Hospital of Parma, Parma, Italy; ^11^ Department of Pathology, Fondazione IRCCS Istituto Nazionale dei Tumori, Milan, Italy; ^12^ Department of Oncology and Hemato-Oncology, University of Milan, Milan, Italy

## Abstract

Under common therapeutic regimens, the prognosis of human papillomavirus (HPV)–positive squamous oropharyngeal carcinomas (OPCs) is more favorable than HPV-negative OPCs. However, the prognosis of some tumors is dismal, and validated prognostic factors are missing in clinical practice. The present work aimed to validate the prognostic significance of our published three-cluster model and to compare its prognostic value with those of the 8^th^ edition of the tumor-node-metastasis staging system (TNM8) and published signatures and clustering models.

**METHODS:**

Patients with HPV DNA-positive OPCs with locoregionally advanced nonmetastatic disease treated with curative intent (BD2Decide observational study, NCT02832102) were considered as validation cohort. Patients were treated in seven European centers, with expertise in the multidisciplinary management of patients with head and neck cancer. The median follow-up was 46.2 months (95% CI, 41.2 to 50), and data collection was concluded in September 2019. The primary end point of this study was overall survival (OS). Three-clustering models and seven prognostic signatures were compared with our three-cluster model.

**RESULTS:**

The study population consisted of 235 patients. The three-cluster model confirmed its prognostic value. Two-year OS in each cluster was 100% in the low-risk cluster, 96.6% in the intermediate-risk cluster, and 86.3% in the high-risk cluster (*P* = .00074). For the high-risk cluster, we observed an area under the curve = 0.832 for 2-year OS, significantly outperforming TNM 8th edition (area under the curve = 0.596), and functional and biological differences were identified for each cluster.

**CONCLUSION:**

The rigorous clinical selection of the cases included in this study confirmed the robustness of our three-cluster model in HPV-positive OPCs. The prognostic value was found to be independent and superior compared with TNM8. The next step includes the translation of the three-cluster model in clinical practice. This could open the way to future exploration of already available therapies in HPV-positive OPCs tailoring de-escalation or intensification according to the three-cluster model.

## INTRODUCTION

Among head and neck squamous cell carcinomas (HNSCCs), the incidence of oropharyngeal cancer (OPC) has been increasing in the past few decades. This is mainly due to the increasing number of human papillomavirus (HPV)–related OPCs.^1^ The first findings of HPV infection in HNSCC have been reported by European research groups in the 1980s,^2^ followed by a first causal association by expression analysis and a link to the OPC in the 1990s.^3^ The association between HPV-positive OPC and the positive prognostic impact has been described approximately 10 years later.^4^ The favorable prognosis of patients with HPV-positive OPC led to explore treatment de-escalation in recent clinical trials. However, expectations were in contrast with the results because this approach was associated with poorer outcomes. Thus, standard of care has not been changed.^5,6^ Therefore, there is still a strong need for trustable prognostic factors.^7,8^ Different preclinical and clinical studies have been performed leading to the identification of several genomic, epigenomic, transcriptomic, and proteomic characteristics of HPV-positive OPCs.^9-14^ At present, the most relevant clinical prognostic factors are stage, extranodal extension (ENE), and smoking exposure.^15^ ENE is currently part of the AJCC and UICC staging system for HPV-negative HNSCCs, but not for HPV-positive ones. In particular, in p16-negative OPC and non-OPC HNSCCs, clinical or radiologic ENE is considered for cN staging, and histopathologic ENE for pN one. Nonetheless, its negative prognostic impact was found in HPV-positive OPC as well, so some authors suggested its implementation in refining the cN of the 8^th^ edition of the tumor-node-metastasis staging system (TNM8).^16^ In the field of gene expression (GE) profiling, the pioneering studies of Slebos^9^ and Pyeon^17^ (referred to the initial characterization of potential biomarkers able to differentiate HPV-positive from HPV-negative HNSCC) and numerous omics characteristics of HPV-positive OPCs were identified in preclinical and clinical studies.^9-14^ Additionally, the analysis of HNSCC transcriptomic data highlighted the existence of HPV-positive distinctive prognostic clusters. In particular, we published a meta-analysis of publicly available transcriptomic data sets of HPV-positive HNSCC, in which three different clusters were characterized. Our three-cluster model was commented and compared with previous HPV-positive HNSCC subtypes in a recent comprehensive review.^18^ Moreover, Dhawan^19^ performed a meta-analysis of OPC data sets with available survival data to determine whether currently available GE signatures could prognosticate outcome more accurately than established clinicopathologic predictors.

**CONTEXT**

**Key Objective**
To validate the prognostic value of a three-cluster gene expression model for human papillomavirus (HPV)–related oropharyngeal squamous cell carcinoma in an independent multicenter case material included in the BD2Decide observational study (NCT02832102).
**Knowledge Generated**
The stratification of the proposed three-cluster model confirmed its prognostic value, significantly outperforming the 8^th^ edition of the tumor-node-metastasis staging system and other available transcriptomic signatures. The three clusters also differed for their biological peculiarities. The biological characterization highlighted the differential expression of key components of the tumor microenvironment as potentially targetable pathways.
**Relevance**
This study confirmed the presence of three different prognostic and biological clusters for HPV-positive oropharyngeal squamous cell carcinoma. Moreover, it supported the potential of introducing a gene expression model in HPV-related oropharyngeal carcinoma to provide a valuable biological basis for the design of clinical studies in the context of treatment tailoring.

Exploiting a well-characterized homogenous HPV-positive OPC cohort with locoregionally advanced disease treated with curative intent,^20^ the present study aims to validate the prognostic significance of our previously identified three-cluster model and to compare its prognostic value with those of TNM8 and the most relevant already published signatures and clustering models.

## METHODS

### Patient Selection

Patients with HNSCC with locoregionally advanced disease (stages III and IV according to TNM 7th edition^21^) and treated with curative intent had been included in the project Big Data and Models for Personalized Head and Neck Cancer Decision Support (BD2Decide).^20^ The study was approved by institutional review boards and ethical committee in March 2016, and patients consented to enrollment. The study population included 1,537 patients diagnosed and treated between 2008 and 2017, and the follow-up closed in September 2019. In this work, data and analyses are focused on patients with HPV-positive OPC. To reduce confounding variables affecting oncologic outcome and consequentially prognostic accuracy of the proposed model, we excluded (1) patients with p16-positive but HPV DNA-negative OPC^22^ and (2) patients receiving unimodal treatment (surgery without postoperative radiation or exclusive radiotherapy without concomitant systemic treatments) for clinical stage T1N > 1, T2N > 1, and T3-T4 any N disease.

### HPV Status and Transcriptomic Experiments

Standard HPV testing was performed on OPC samples by p16 immunohistochemistry, followed by, in case of p16-positive immunostaining, a HPV DNA test.^20^

In case of polymerase chain reaction (PCR), p16-positive cases were tested using the GP5+/6+ DNA PCR assay, aimed at detecting 14 high-risk HPV types (ie, HPV 16, 18, 31, 33, 35, 39, 45, 51, 52, 56, 58, 59, 66, and 68). If DNA PCR was negative, HPV16 E7 primers were used to exclude L1 integrations.

In case of in situ hybridization, p16-positive OPC specimens were tested using the INFORM HPV III Family 16 Probe, able to identify 10 high-risk genotypes (ie, HPV 16, 18, 31, 33, 35, 45, 52, 56, 58, and 66). When the DNA was not evaluable through in situ hybridization, the E6/E7 mRNA of high-risk HPV was assessed through the RNA scope Probe HPV HR18, identifying HPV 16, 18, 26, 31, 33, 35, 39, 45, 51, 52, 53, 56, 58, 59, 66, 68, 73, and 82.

Positive results in both p16 immunohistochemistry and HPV DNA testing were considered evidence of a transcriptionally active HPV infection.^23^ GE data sets had been generated using Affymetrix human Clariom D microarrays (Affymetrix, Santa Clara, CA).^20^ MIAME-compliant data are deposited in the GEO repository (GSE163173).

### Bioinformatics Analysis

After microarray data normalization, the HPV-positive OPC selected cohort was classified according to the three-cluster model.^24^ Tumor microenvironment, the immune and stroma scores, and other cell components were inferred by xCell.^25^ Pairwise gene set enrichment analysis (GSEA)^26^ comparisons were performed to discern which Hallmark gene sets^27^ were differentially enriched. In the final data set, we assessed the overall survival (OS) prognostic discrimination ability of our three-cluster model by area under the curve (AUC) at 2 years, and its performance was challenged against TNM8 and publicly available prognostic models and signatures:

(A) HPV-positive OPC signatures: (A1) Gleber-Netto^28^ and (A2) Chen^29^;
(B) HNSCC signatures: (B1) De Cecco 172 genes^30^ and (B2) De Cecco Hypoxia^10^ signature able to identify aggressive tumors; and
(C) multiple cancers signatures: (C1) Torres-Roca RSI^31^ directly proportional to radioresistance and (C2) Buffa Hypoxia.^32^ Moreover, three already described signatures (Slebos-up, Pyeon-up, and Zhang), clustering HPV-positive OPC, were applied to our final data set and compared with our three-cluster model. Three signatures (radiosensitivity index [RSI], De Cecco Hypoxia, and De Cecco 172 genes) were applied following the linear model provided in the original studies. Regarding the other models, a metagene approach was applied: A score was computed in our data set by ssGSEA using the GSVA R package^33^ providing an unsupervised method for estimating variation of gene set enrichment through the samples. AUC at 2 years was assessed through timeROC R package.^34^

To enable a better understanding of strengths and limits of the available signatures, studies and their characteristics are summarized in the Data Supplement.

### Statistical Analysis

Differences between the survival curves of the three clusters were assessed using the log-rank test and R package survival. Primary end point was OS, and secondary end point was disease-free survival (DFS). For survival analyses, we set 2 years of observation. Further details are provided in the Data Supplement.

## RESULTS

### Definition of the Final Data Set

**FIG 1.**
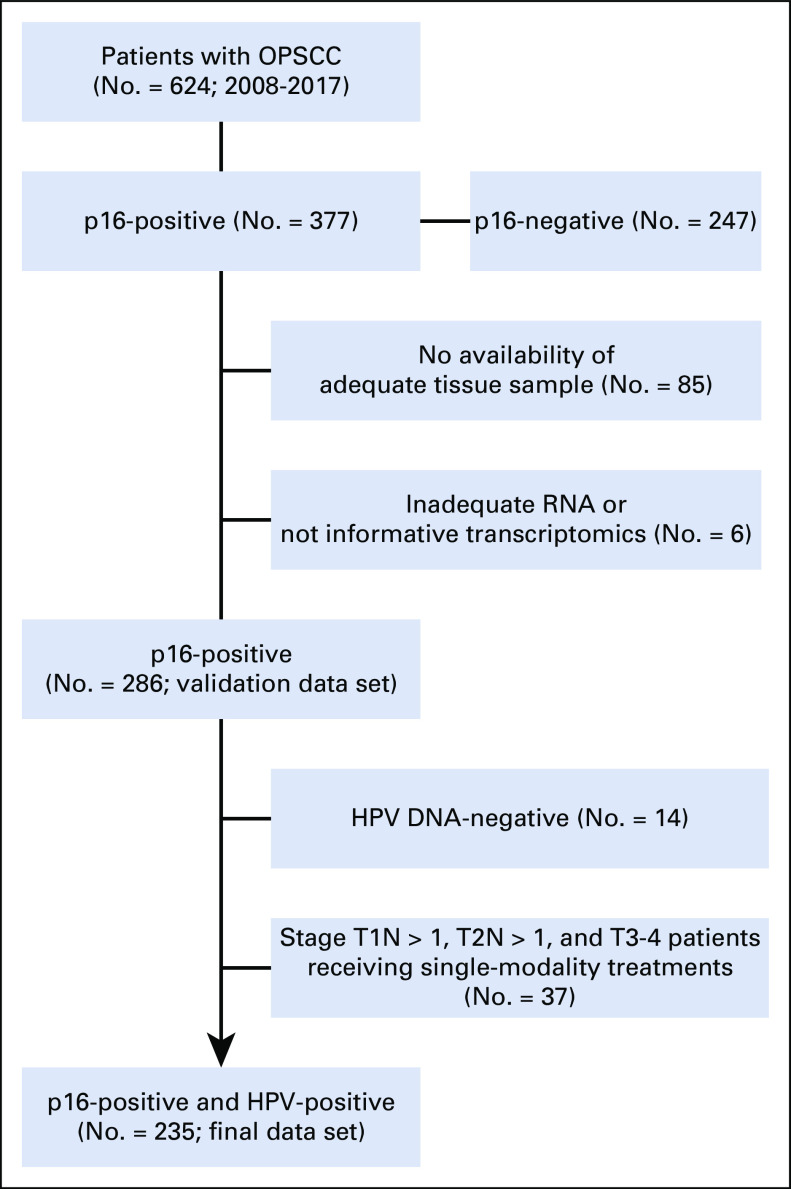
Selection of the final data set. HPV, human papillomavirus; OPSCC, oropharyngeal squamous cell carcinoma.

In the BD2Decide project, 624 patients with stage III-IV (TNM 7th edition) OPC were included, and 377 patients were p16-positive. For validation analysis, we analyzed the cohort composed of 286 p16-positive cases with informative transcriptomics. Among them, 235 p16-positive and HPV-positive cases (14 excluded because of HPV DNA-negative and 37 excluded because of single-modality treatments) were suitable as a final data set for clinical correlations (Fig 1). No significant differences were recorded between the cohorts (Data Supplement). In the final data set, the median follow-up was 46.2 months (95% CI, 41.2 to 50), 79% of patients were men (185), and the median age was 60 years (Table 1). Of the 225 cases with regional lymph node involvement, 18% had a radiologic and/or clinical ENE, defined as the radiologic and/or clinical evidence of cancer invasion through nodal capsule or beyond.^35^ Two thirds of patients were current or previous smokers at diagnosis, and 71% of them had a tobacco exposure of > 10 pack-years. According to the prognostic stratification of 36 from the work of Ang et al, 68% of patients with HPV-positive OPCs had a low-risk disease and the remaining 32% of cases were at intermediate risk (74 patients; unknown in one case). According to the current TNM staging system (TNM8), disease stage was I in 48% of cases (112 patients), II in 27% (64 patients), and III in 25% (59 patients). By applying the three-cluster model to the 235 data set, the following distribution was observed: low risk or Cl1, 108 (46%) cases; high risk or Cl2, 30 (13%) cases; and intermediate risk or Cl3 97 (41%) cases. The clinical characteristics and treatment modalities were not significantly different, with the exception of the frequency of cases with ENE-positive regional lymph node metastases: low risk 12.5%, high risk 37%, and intermediate risk 18% (*P* = .012; Table 1).

**TABLE 1.**
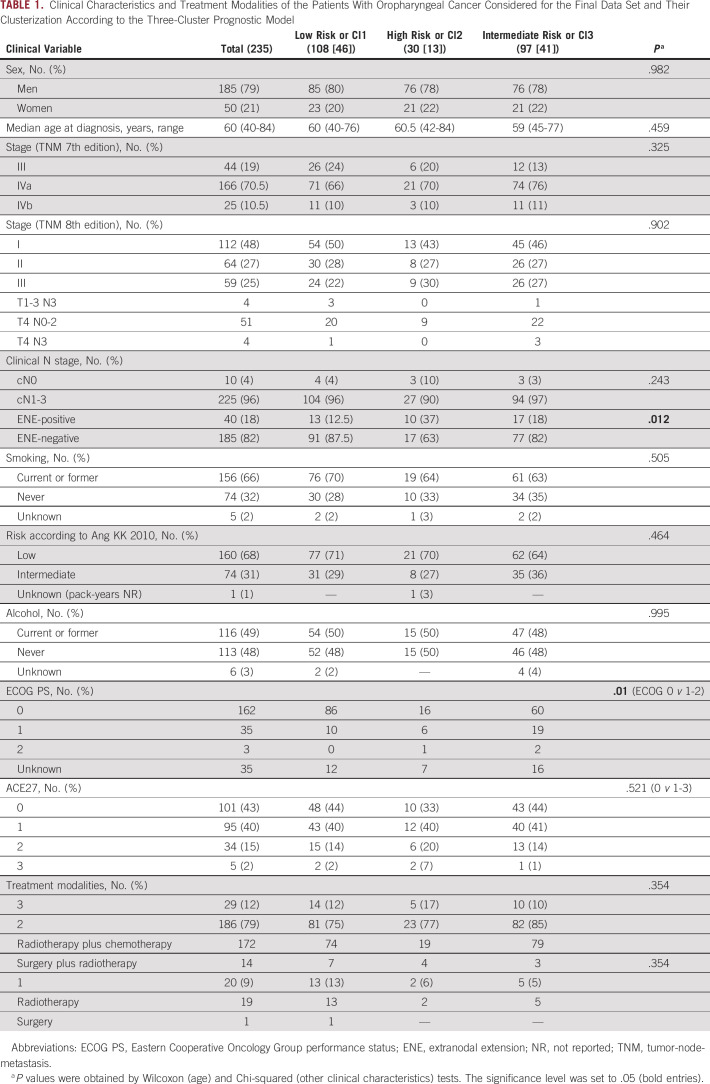
Clinical Characteristics and Treatment Modalities of the Patients With Oropharyngeal Cancer Considered for the Final Data Set and Their Clusterization According to the Three-Cluster Prognostic Model

### Prognostic Analysis

**FIG 2.**
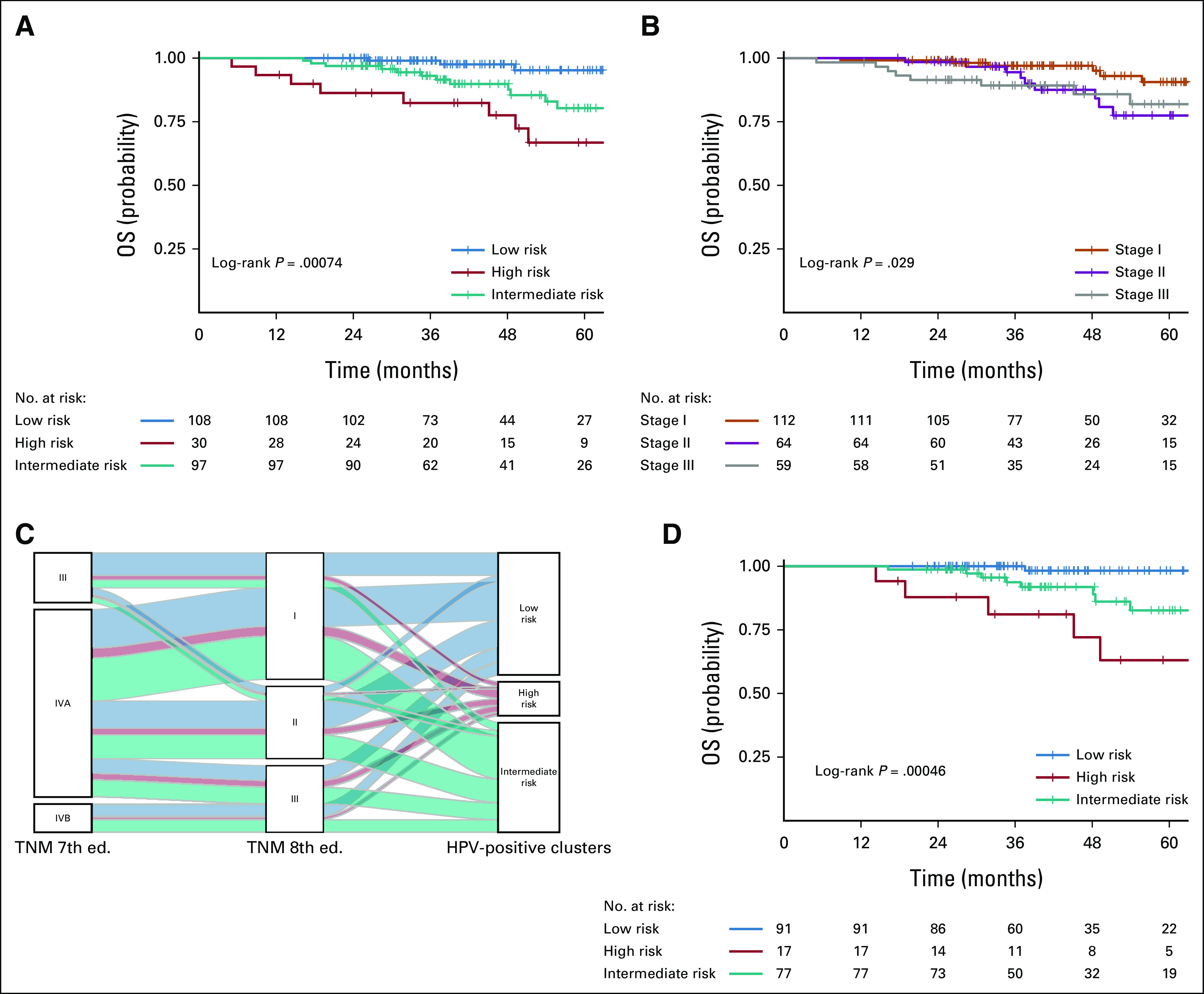
Prognostic analysis of the final data set (n = 235): (A) OS evaluation according to the three-cluster model; (B) OS according to TNM, 8th edition; (C) Alluvial diagram; and (D) OS of the 185 ENE-negative cases. ENE, extranodal extension; OS, overall survival; TNM, tumor-node-metastasis.

The prognostic stratification according to the three-cluster model was tested in both the validation (Data Supplement) and final data sets (Fig 2A) and confirmed the rank order of the three-cluster model obtained in the original meta-analysis (Data Supplement).^24^ The 2-year OS differed among the three clusters (low risk 100%, high risk 86.3%, and intermediate risk 96.9%; *P* = .00074). Considering low risk as a reference group, hazard ratio for death was 9.15 (95% CI, 2.42 to 34.5; *P* = .001) for high versus low risk and 5.67 (95% CI, 1.64 to 19.6; *P* = .006) for intermediate versus low risk. AUC for 2-year OS was 0.832 (Data Supplement). Considering 2-year OS, AUC was 0.754 for TNM8 versus 0.596 for TNM7 (Data Supplement).

Survival and recurrence probability were predicted with statistical significance by TNM8 (Fig 2B), not by TNM7 (Data Supplement). Through an alluvial plot, the distribution of patients according to the TNM7 and TNM8 classification and the three clusters was explored (Fig 2C; Data Supplement). Table 2 reports the OS prognostic power at 2 years for TNM8 (AUC 0.754) and seven publicly available prognostic signatures. Besides the three-cluster model, three signatures (two hypoxia signatures^32^ and RSI^31^) outperformed TNM8 and were able to significantly separate high-risk versus low-risk HPV-positive OPC cases. Box plot distribution (Data Supplement), according to the scores of the seven selected prognostic signatures in each of the three clusters, confirmed that the high risk exhibited significantly the highest RSI and hypoxia expression. The clustering was not influenced by TNM staging also at multivariate analysis (OS: three-cluster model *P* = .0056 *v* TNM8 *P* = .073; DFS: three-cluster model *P* = .0008 *v* TNM8 *P* = .031), and the combination of three-cluster model and TNM8 provided AUC = 0.842 for OS and AUC = 0.809 for DFS (Data Supplement) at 2 years. At bivariate analysis, the three clusters maintained their prognostic power (OS *P* = .009, DFS *P* = .001) independently of ENE status (Fig 2D and Data Supplement). Two-year DFS was 99.1% in low risk, 76.2% in high risk, and 91.8% in intermediate risk (*P* = .0002). Using low risk as reference, hazard ratio for disease recurrence was 6.1 (95% CI, 2.37 to 15.76; *P* = .0002) for high versus low risk and 3.67 (95% CI, 1.57 to 8.59; *P* = .0027) for intermediate versus low risk. AUC for 2-year DFS was 0.777 (Data Supplement). The highest AUCs were observed for the three-cluster model. Ang classification was prognostic for DFS (*P* = .039), not for OS (*P* = .069; Data Supplement).

**TABLE 2.**
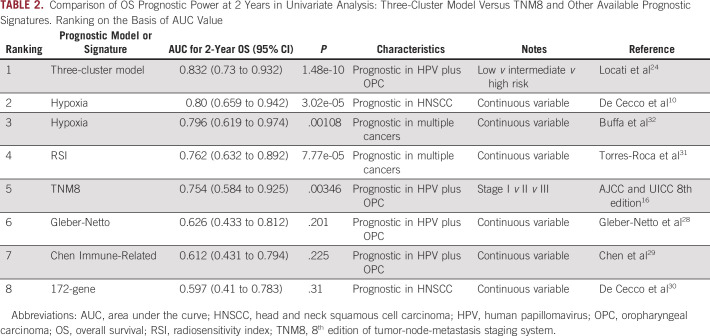
Comparison of OS Prognostic Power at 2 Years in Univariate Analysis: Three-Cluster Model Versus TNM8 and Other Available Prognostic Signatures. Ranking on the Basis of AUC Value

### Tumor Biological Landscape

**FIG 3.**
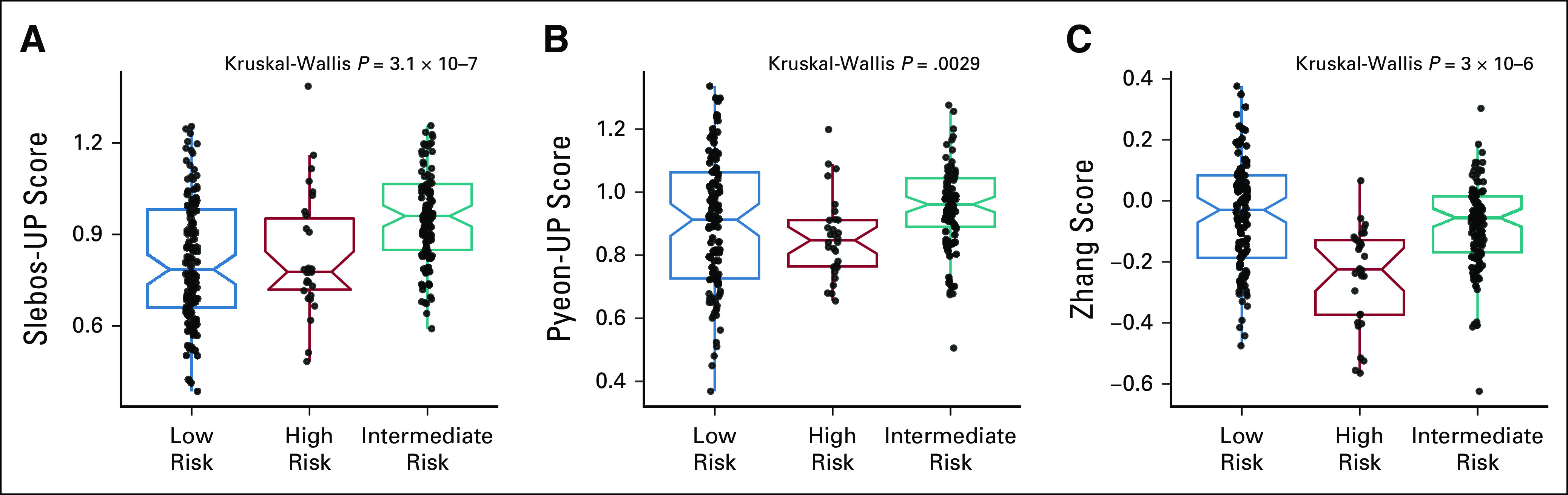
HPV-clustering models applied to the final data set subdivided in the three-cluster model: (A) box plot of Slebos-UP score, (B) box plot of Pyeon-UP score, and (C) box plot of Zhang score. HPV, human papillomavirus.

**FIG 4.**
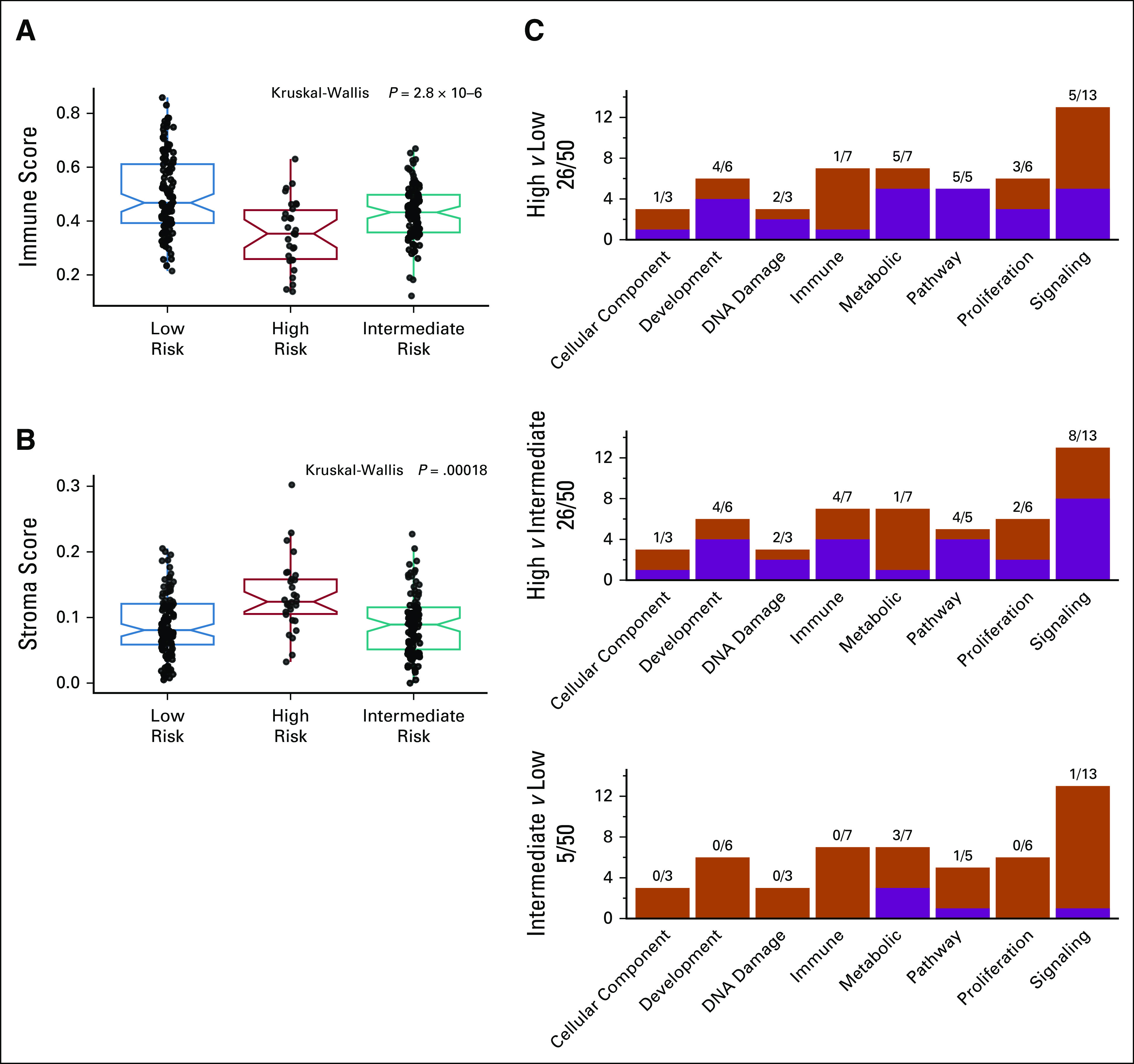
Immune or functional characterization of the three clusters by xCell and Hallmarks: (A) box plot of immune score, (B) box plot of stroma score, and (C) summary of enriched Hallmark gene set for the three-cluster pairwise comparisons.

Three signatures reported molecular patterns associated with HPV status;^9,17,37^ these signatures and their performance in stratifying HPV tumors were investigated and compared with our three-cluster model (Fig 3). The stratification ability of pioneering signatures^9,17^ was not confirmed because of the limited sample size (Fig 3A and Fig 3B). On the other hand, a significant difference was observed with Zhang clustering (Fig 3C), and the distribution in the three clusters confirmed the similarity, as already highlighted in the review by Qin et al.^18^ The samples of the final data set, subdivided according to the three-cluster model, were analyzed for difference in cell composition by xCell bioinformatics tool (Data Supplement), essentially confirming the results obtained in the original study.^24^ The highest level of immune score was recorded in low risk (*P* = 2.8 × 10^−6^; Fig 4A). When the immune populations were explored in detail, we observed a significant enrichment of total or memory CD4 T cells, effector memory CD8 T cells, and B cells in low risk, followed by intermediate risk and high risk (Data Supplement). The highest level of stroma score was reached in high risk (*P* = .00018, Fig 4B); the detailed analysis of the microenvironment revealed an abundance in fibroblasts, keratinocytes, and endothelial cells in high risk, while epithelial cells characterized both high and intermediate risks (Data Supplement). To gain a deeper insight into the biological and functional differences among the three clusters, analysis of the 50 hallmark gene sets in GSEA was performed in the final data set (Fig 4C). According to GSEA, high risk exhibited a strong enrichment of 26 of 50 hallmark gene sets versus low and intermediate risk (Data Supplement), and 18 gene sets were in common. In particular, we observed enrichment: in high versus low risk, in epithelial mesenchymal transition (development), glycolysis (metabolism), UV response (DNA damage), NOTCH signaling, TGF beta signaling, hypoxia (pathway), mitotic spindle, p53 pathway, and MYC target V1 (proliferation); in intermediate versus low risk in metabolic activation; and in common in high and intermediate versus low risk, in MTORC1 (signaling), reactive oxygen species (pathway), cholesterol homeostasis, and xenobiotic metabolism (metabolism). Sixteen GSEA pathways are characterized by immune-exhausted HNSCCs. Among them, 38 pathways were overexpressed in high risk, 15 in intermediate risk, and 10 in low risk (Data Supplement).

## DISCUSSION

This is an independent validation of a three-cluster model, previously developed through meta-analysis of publicly available data sets.^24^ The main difference between the original and present data sets relies on the current study cohort. Our original meta-analysis^24^ had a limitation since the cases were HNSCC HPV-positive, but 32% of them were non-OPC. The present analysis considered only HPV-positive locoregionally advanced OPC patients treated with curative intent. All the HPV-positive not OPCs together with HPV DNA-negative or suboptimally treated patients were excluded from the final data set. This homogeneous patient series and the availability of controlled clinical and pathologic data led to the refinement of the three-cluster model on the largest data set of OPC presently available. Unsupervised clustering analysis is a popular approach to discover novel biological-oriented disease subtypes. This approach has deeply been applied in breast cancer starting from the early 2000s; the signature was refined and its last version, PAM50, added prognostic value to the traditional pathologic, histologic, and biological parameters.^38^ Clustering models of HNSCC associated with HPV have been already developed essentially defining two clusters associated with HPV-positive tumors. However, these studies^28,37,39^ contained non-OPC HPV-positive HNSCCs and HPV-negative OPCs. In the original meta-analysis,^24^ we observed that most of the HPV-positive non-OPC cases belonged to the high-risk cluster. It is noteworthy that the majority of patients (47%, 24 of 51) excluded from the validation set, because of HPV DNA-negative or suboptimally treated, were part of the high-risk cluster. Thus, the results obtained on OPC final data set indicated that, after removal of potential confounding factors, high risk maintained the worst prognosis. The present work definitely confirmed the prognostic value of the three-cluster model in HPV-positive OPCs. The prognostic accuracy of the three-cluster model (AUC = 0.832 for 2-year OS) outperformed the TNM8, the current gold standard for prognostic stratification in oncology, and at bivariate analysis, its prognostic role was independent. In addition to the three-cluster model, three prognostic signatures, even if developed in all sites HNSCC or multiple cancers or OPC HPV-negative cases, were also able to outperform TNM8. They significantly separate the OPC HPV-positive cases with an AUC > 0.76. Interestingly, these signatures were obtained by meta-analysis of more than a 1,000 samples and/or were validated in different published studies. On the contrary, the other three signatures and models, built on smaller or heterogeneous HNSCC cohorts, did not show a prognostic role, suggesting the importance of larger or homogeneous study design in the context of the omics studies. The prognostic performance of the three-cluster model was not influenced by underlying clinical factors. The only difference in terms of clinical characteristics among the three clusters was ENE prevalence. Indeed, high-risk patients had a higher frequency of ENE plus disease, reflecting a more aggressive disease.^35^ This consistent association corroborates the robust independent prognostic ability of the proposed three-cluster model. Low risk was characterized by the best prognosis and included 46% of patients. These tumors expressed a high immune score and significant enrichment of immune active cells (total or memory CD4 T cells, effector memory CD8 T cells, and B cells). They were characterized by a low RSI score and the lowest hypoxia scores. Our low-risk cluster is probably similar to the HPV-IMU identified by Zhang et al,^37^ also confirmed by the experimental comparison of Qin et al.^18^ The worst prognosis was observed in high-risk patients (13% of the study cohort). This group, characterized by the highest keratinocytic differentiation status, is similar to the HPV-KRT cluster of Zhang et al^37^ and was confirmed in the comparison of Qin et al.^18^ Moreover, the high-risk cluster was characterized by the lowest immune score and an enrichment in GSEA pathways associated with the immune-exhausted HNSCC tumors,^38^ which is also defined as cold immune.^18^ The enrichment in hypoxia hallmark, consistent with the immune suppression and radioresistance (high RSI), was confirmed by the highest expression of hypoxia signatures.

The novelty introduced by our work was the identification of the intermediate-risk cluster (41% of the study cohort), never characterized before by other clustering models.^14-16^ Despite our efforts, this was the less biologically and functionally characterized cluster. Nevertheless, biologically we were able to separate this group from the patients with good (low risk) or poor (high risk) prognosis. In detail, it expressed an intermediate immune score not associated with exhausted immune cells, a marginal radioresistance and low expression of De Cecco hypoxia signature. Notably, we observed an evident enrichment in metabolic pathways in both high and intermediate risks. Further investigation and deeper comments about possible treatments applicable to patients with HPV-positive OPC are reported in the review of Qin et al^18^ on the basis of the experimental comparison of the three-cluster model^24^ data with the other published HPV-positive two-cluster models.^14,17,28,37,39^ In addition, we observed that in our study population, the majority of current or previous smokers had a relevant tobacco exposure (> 10 pack-years in 71% of cases). Nevertheless, the frequency of current or previous smokers in our study population (66%) was lower than the one reported in previously published studies (82% in the RTOG0129 trial^36^). This is consistent with the observed smoking reduction in Western countries, where cigarette smoking rates have been reducing in the past 50 years. Moreover, future projections are forecasting a further reduction in smoking prevalence.^40^ These epidemiologic variations imply that the observations found in our study population might need further updates in the next decades, to study whether behavioral changes will be reflected by different biological alterations, with possible clinical impact. Potential limitations of the three-cluster model are the observed dropout rate (25%) in our GE case material compared with the original clinical set and the lack of specification of the viral genotype for each case. The first limitation could be due to the fact that for most OPC cases, where chemoradiation is usually preferred over surgery, the analyzed specimen was an initial small biopsy. However, independent studies show that an increase in sample size, corresponding to our dropout rate, had limited effect on the clustering performance.^37^ The second drawback could be attributed to the HPV DNA tests used. However, this limitation is mitigated by the fact that the vast majority of HPV-positive OPCs are known to be related to the HPV16 genotype.^41^ Indeed, we do not anticipate major negative impacts of the cited drawbacks on our analysis.

In conclusion, in patients with HPV-positive OPC with locoregionally advanced disease treated with curative intent, there is still a strong need for defining trustable prognostic factors, and our results seem to fulfill this necessity. The rigorous clinical selection of the cases included in the BD2Decide study corroborates the robustness of the proposed three-cluster model, which resulted in an independent and superior prognostic factor compared with TNM8, the current gold standard for prognostic forecasting in clinical practice. The next step includes the required translation of this GE clustering in clinical practice, following a framework applied in other tumors. This could open the way to future exploration of already available therapies in HPV-positive OPCs tailoring de-escalation or intensification according to the three-cluster model.
